# Fine microstructure formation in steel under ultrafast heating and cooling

**DOI:** 10.1038/s41598-022-06280-x

**Published:** 2022-02-09

**Authors:** Mitsuharu Yonemura, Hitomi Nishibata, Rina Fujimura, Natsumi Ooura, Kengo Hata, Kazuki Fujiwara, Kaori Kawano, Itsuki Yamaguchi, Tomoyuki Terai, Yuichi Inubushi, Ichiro Inoue, Toshinori Yabuuchi, Kensuke Tono, Makina Yabashi

**Affiliations:** 1 Advanced Technology Research Laboratories, Nippon Steel Corporation, 1-8 Fuso-cho, Amagasaki, Hyogo 660-0891 Japan; 2 Hanshin Unit Osaka Testing Div., Nippon Steel Technology Corporation, 5-1-109 Shimaya, Osaka, 554-0024 Japan; 3 Department of Materials Science and Engineering, Graduate School of Engineering, Osaka University, 2-1 Yamadaoka, Suita, Osaka 565-0871 Japan; 4 Japan Synchrotron Radiation Research Institute, 1-1-1 Kouto Sayo-cho Sayo-gun, Hyogo, 679-5198 Japan; 5 RIKEN SPring-8 Center, 1-1-1 Kouto Sayo-cho Sayo-gun, Hyogo, 679-5148 Japan

**Keywords:** Structural materials, Techniques and instrumentation

## Abstract

This study evaluates phase transformation kinetics under ultrafast cooling using femtosecond X-ray diffraction for the operand measurements of the dislocation densities in Fe–0.1 mass% C–2.0 mass% Mn martensitic steel. To identify the phase transformation mechanism from austenite (γ) to martensite (α′), we used an X-ray free-electron laser and ultrafast heating and cooling techniques. A maximum cooling rate of 4.0 × 10^3^ °C s^–1^ was achieved using a gas spraying technique, which is applied immediately after ultrafast heating of the sample to 1200 °C at a rate of 1.2 × 10^4^ °C s^–1^. The cooling rate was sufficient to avoid bainitic transformation, and the transformation during ultrafast cooling was successfully observed. Our results showed that the cooling rate affected the dislocation density of the γ phase at high temperatures, resulting in the formation of a retained γ owing to ultrafast cooling. It was discovered that Fe–0.1 mass% C–2.0 mass% Mn martensitic steels may be in an intermediate phase during the phase transformation from face-centered-cubic γ to body-centered-cubic α′ during ultrafast cooling and that lattice softening occurred in carbon steel immediately above the martensitic-transformation starting temperature. These findings will be beneficial in the study, development, and industrial utilization of functional steels.

## Introduction

High-strength low-alloy steels are the most widely used materials in automotive and construction industries for improving the fuel efficiency of automobiles and ensuring high strength and safety features of buildings. The heat treatment of steel involves several processes (e.g., stress relieving, normalizing, and annealing) to condition the microstructure and obtain the desired mechanical properties. The metallurgy of steel produced using hot rolling and subsequent accelerated cooling in hot strip and plate mills as a thermomechanical controlled process has been investigated for several decades^1^, and the formation of ultra-fine grains during production via cooling rate control has been extensively investigated^2–4^. Martensitic transformation, which controls the mechanical properties of metals, is a vital phenomenon for ensuring high strength.

Several studies have focused on improving both the strength and ductility of low-carbon steels through grain refinement via rapid heating^5–10^ and rapid cooling^11–13^. Petrov^14,15^ reported that the average grain size decreased from 5 to 1 μm as the heating rate increased from 10^2^ to 10^3^ °C s^−1^ in cold-rolled high-strength low-alloy steels and dual-phase steels, increasing the tensile strength. In a previous study^16^, a maximum heating rate of 1.2 × 10^4^ °C s^−1^, which is sufficient to avoid diffusive reversion, was achieved, and the reverse transformation during ultrafast heating was successfully observed. The results demonstrated that a fine microstructure was formed due to a phase transformation, in which the dislocation density and carbon concentrations remained high owing to ultrafast heating. We speculated that relatively finer grains without fabrication might surpass the category of conventional steels. The final microstructure of the steel depends significantly on the cooling rate to which the plate or strip is subjected immediately after rolling. Ultrafast cooling technology is a new cooling mode developed in recent years^17–20^ that can refine grains and improve the strength of steel. Therefore, the combination of ultrafast heating and cooling without soaking time results in the formation of martensite, bainite, and retained austenite phases^21^. Moreover, researchers observed a hexagonal closed-pack (HCP) structure in the intermediate phase during the transformation from a face-centered-cubic (FCC) austenite (γ) to a body-centered-cubic (BCC) martensite (α′)^22,23^. The HCP phase of iron does not appear at ambient pressure but forms under a pressure of approximately 11 GPa in pure iron^22^. The stabilization of the HCP phase via internal stresses under rapid cooling has been reported^23^. Despite these studies and other extensive efforts, a complete understanding of microstructural changes under ultrafast cooling (i.e., cooling faster than 10^3^ °C s^–1^) following ultrafast heating (i.e., heating faster than 10^4^ °C s^–1^) has remained elusive due to the lack of operand measurements that can provide information regarding martensitic transformations. To the best of our knowledge, microstructural changes under ultrafast cooling have not been directly observed to date.

Herein, we discuss the effects of ultrafast cooling following ultrafast heating on microstructural formation within the context of dislocation migration using femtosecond X-ray diffraction for the operand measurements of the dislocation densities in Fe–0.1 mass% C–2.0 mass% Mn martensitic steel.

## Methods

### Experimental setup

Since the measurement technique is surface sensitive, some steps were taken to ensure similar surface qualities between the specimens as follows. The scale and decarburization layers from hot rolling were removed by mechanical grinding, and the subsequent heat treatment for martensitization was performed in a reducing atmosphere. The scale from this heat treatment was removed by pickling, and the sample was cold-rolled to reduce its thickness by 50%. Further, by checking the X-ray diffraction patterns before heating, the reproducibility of the measurements between the specimens was ensured. The chemical component after cold working was 0.1 mass% C–1.97 mass% Mn–0.06 mass% Si–0.047 mass% P–0.001 mass% S–0.014 mass% Al–0.0011 mass% N–bal. Fe. Single-shot X-ray diffraction (XRD) measurements are highly effective for observing rapid and irreversible microstructural changes. Synchrotron radiation facilities produce such bright X-rays, and diffraction patterns have been recorded with exposure times as short as approximately 10 ms^24,25^. However, such exposure times are significantly longer than those of target temporal resolutions < 1 μs for the direct observation of rapid microstructural changes in iron and steel. Therefore, more intense X-ray beams are required, with the flux densities increased by at least a factor of 10^4^. Currently, these types of intense X-ray beams can be produced using an X-ray free-electron laser (XFEL)^26^.

In this study, time-resolved XRD measurements were performed using an XFEL from the SPring-8 Angstrom Compact Free Electron Laser (SACLA) (Hyogo, Japan)^27,28^ to clarify the changes in the dislocation densities during the martensitic transformations at ultrafast cooling rates up to and exceeding 10^3^ °C s^–1^. This rate is much higher than that reported for previous operand techniques^24,25^ and enables hitherto an unexplored regime of non-equilibrium states to be investigated.

**Figure 1:**
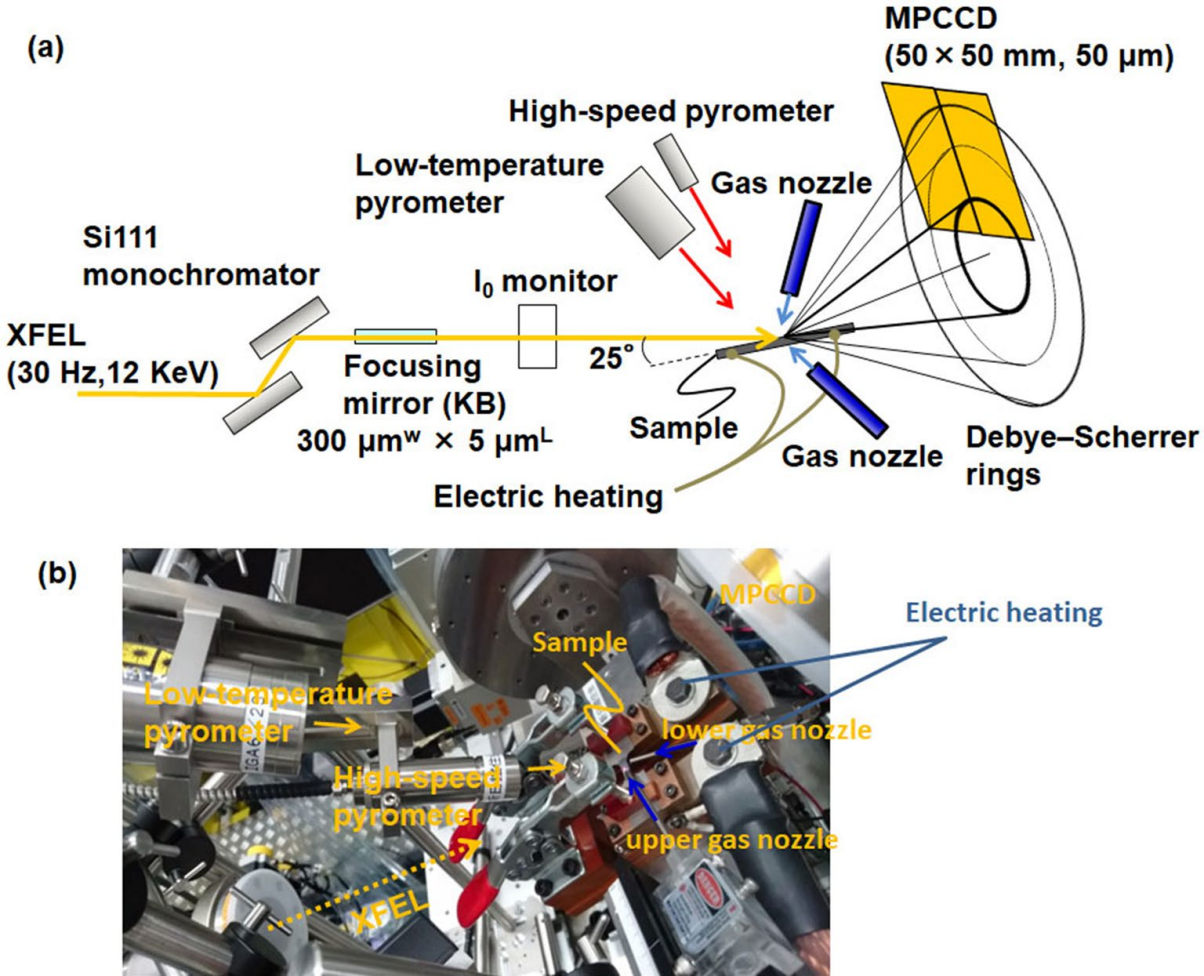
Experimental setup for operand measurement during ultrafast heating and ultrafast cooling. (**a**) Schematic illustration and (**b**) photograph around the sample.

To clarify the phase transformations during the ultrafast cooling following the ultrafast heating, a femtosecond XRD was performed using an XFEL. The experimental setup is shown in Fig. 1. The incident beam used was monochromatized with a Si(111) monochromator to achieve an energy width of approximately 1 eV (full width at half maximum (FWHM)). The beam was focused with an elliptical mirror measuring 300 × 7 μm (horizontal × vertical direction). The divergence angle in the vertical direction was 0.1 mrad and the glancing angle to the sample surface was 25°. Photon energy of 12 keV with a wavelength of 0.10 nm was used to measure a sufficient number of diffraction peaks for the α′ and γ phases without any overlaps. For ultrafast electrical heating, temperature measurements were performed using a high-speed pyrometer, whose emissivity was corrected to 0.9 via simultaneous measurements using a type-R thermocouple and a two-color pyrometer at a low heating rate, with a time resolution of 10 μs and a spot size of 400 μm. Since the wavelength of the pyrometer is within the range of 2.0–2.5 μm, the temperature dependence of the emissivity of iron is low^29^. Therefore, the temperature was measured at a constant emissivity.

X-ray detection was performed using a multiport charge-coupled device (MPCCD)^30^, and the heating and cooling system was controlled via a trigger signal from the SACLA. Both edges of the sample were supported using a copper electrode for electrical heating. The heated area of the sample measured 15 × 5 × 0.5 mm^3^. To suppress the effects of crystal orientation, diffraction patterns were measured in a wide reciprocal lattice space using the MPCCD detector with two sensor modules at a camera length of 150 mm and angles of 35°, 55°, and 77° from the horizontal. Heating and temperature measurement were started by the edge trigger of the transistor-transistor-logic (TTL) level that was delayed from the open gate signal of the XFEL at the setup time. Meanwhile, the diffraction measurement was synchronized with the open gate signal. The TTL was controlled at a nanosecond-level speed, which was higher than that of the heating rate. Furthermore, a helium gas spraying system was designed to inhibit high-temperature oxidation and achieve a high cooling rate. The gas spraying timing at the set temperature was controlled using a pulse circuit.

The XFEL frequency was 30 Hz, which corresponded to a temperature step of 333 °C at a heating rate of 10^4^ °C s^–1^. We achieved a temperature resolution of 100 °C or lower by delaying the timing of the TTL via an open gate signal. The sample was replaced with a new one after every heating and cooling cycle. Eleven sets of measurements, per cooling rate, were performed to ensure the statistical relevance of the data. The timing of the TTL was offset between successive samples to get a more continuous XRD pattern during the heat treatment for a given cooling rate. The agreement of the diffraction patterns before heating for the different samples and the systematic changes in the XRD data during the ultrafast heating and cooling process showed that the results from the different samples were consistent. For each sample, 100 dark images were recorded for background correction. In addition, to calibrate the diffraction angles, 100 diffraction images were obtained before heating. These images were obtained within approximately a 2-s test duration for a 4.0 × 10^3^ °C s^−1^ cooling rate test and at a 1.0 × 10^3^ °C s^−1^ cooling rate.

### X-ray line profile analysis

XRD line profiles were obtained by integrating the diffraction images in the circumferential direction. We applied an X-ray line profile analysis (XLPA), which was originally developed by Williamson and Hall^31^ and Warren and Averbach^32^ in the 1950s. Based on a theory proposed by Ungár et al.^33,34^ in the 1980s, the line profiles were analyzed by considering the following: (1) the effects of anisotropic lattice strains on crystallographic orientations, and (2) the strength of the lattice strains around dislocations. These characteristics were used to deduce the optimal relationship between the dislocation density and X-ray line profiles. Large lattice strains occur in specific crystallographic orientations because the Burgers vector depends on the crystal system^35^. From the modified Williamson–Hall and modified Warren–Averbach^33^ procedures using a mean contrast factor based on the elastic anisotropy, the XLPA is used to evaluate the properties of the substructure, such as the dislocation density, edge/screw dislocation fraction, and dislocation arrangement.

The FWHM of the normalized peaks can be evaluated using the modified Williamson–Hall equation, as expressed in Eq. (1).

1 $$\Delta K=\frac{0.9}{D}+{\left(\frac{\pi {M}^{2}{b}^{2}}{2}\right)}^\frac{1}{2}\cdot {\rho }^\frac{1}{2}K{C}^\frac{1}{2}+O\left({K}^{2}C\right),$$

where $K=2sin\theta /\lambda$, and $\Delta K=2cos\theta \left(\Delta \theta \right)/\lambda$ is the magnitude of the FWHM. $\theta$ is the diffraction angle and $\lambda$ is the X-ray wavelength. $D$, $\rho$, and $b$ are the average particle size, average dislocation density, and Burgers vector, respectively. Both $M$ and $O$ are constants, depending on the effective outer cutoff radius of the dislocations.

The dislocation density can be obtained using the modified Warren–Averbach equation, as expressed in Eq. (2).

2 $$lnA\left(L\right)=ln{A}^{s}\left(L\right)-\rho \cdot \frac{\pi {b}^{2}}{2}\cdot {L}^{2}\cdot ln\left(\frac{{R}_{e}}{L}\right)\cdot \left({K}^{2}C\right)+Q\left({K}^{4}{C}^{2}\right)$$

where $A\left(L\right)$ is the real part of the Fourier coefficients, ${A}^{s}$ is the size Fourier coefficient as defined by Warren, ${R}_{\mathrm{e}}$ is the effective cutoff radius of the dislocations, $Q$ is the second-order terms of ${K}^{2}C$, and $L$ is the Fourier length.

The contrast factor $\overline{C }$ depends on the average contrast factor ${\overline{C} }_{h00}$, the parameter of the lattice index (*h*, *k*, *l*) $H$, and $q$, which in turn depends on the elastic constants of the crystal and the characteristics of the dislocations, that is, the screw/edge fraction in the crystals, as shown in Eq. (3).

3 $$\overline{C }={\overline{C} }_{h00}\left(1-q{H}^{2}\right),$$

where $${H}^{2}=\frac{\left({h}^{2}{k}^{2}+{h}^{2}{l}^{2}+{k}^{2}{l}^{2}\right)}{{\left({h}^{2}+{k}^{2}+{l}^{2}\right)}^{2}}.$$

Using Eqs. (1), (2), and (3), $\rho$ was determined.

To estimate the diffraction angle and FWHM, the asymmetric X-ray line profiles were fitted with a split pseudo-Voigt function^36^. Additionally, geometric errors in the XRD measurements were corrected by comparing the diffraction data for the sample (before heating) with the reference data from the National Institute of Standards and Technology (NIST) standard (660c).

## Results and discussion

### Operand measurements during ultrafast heating and cooling

**Figure 2:**
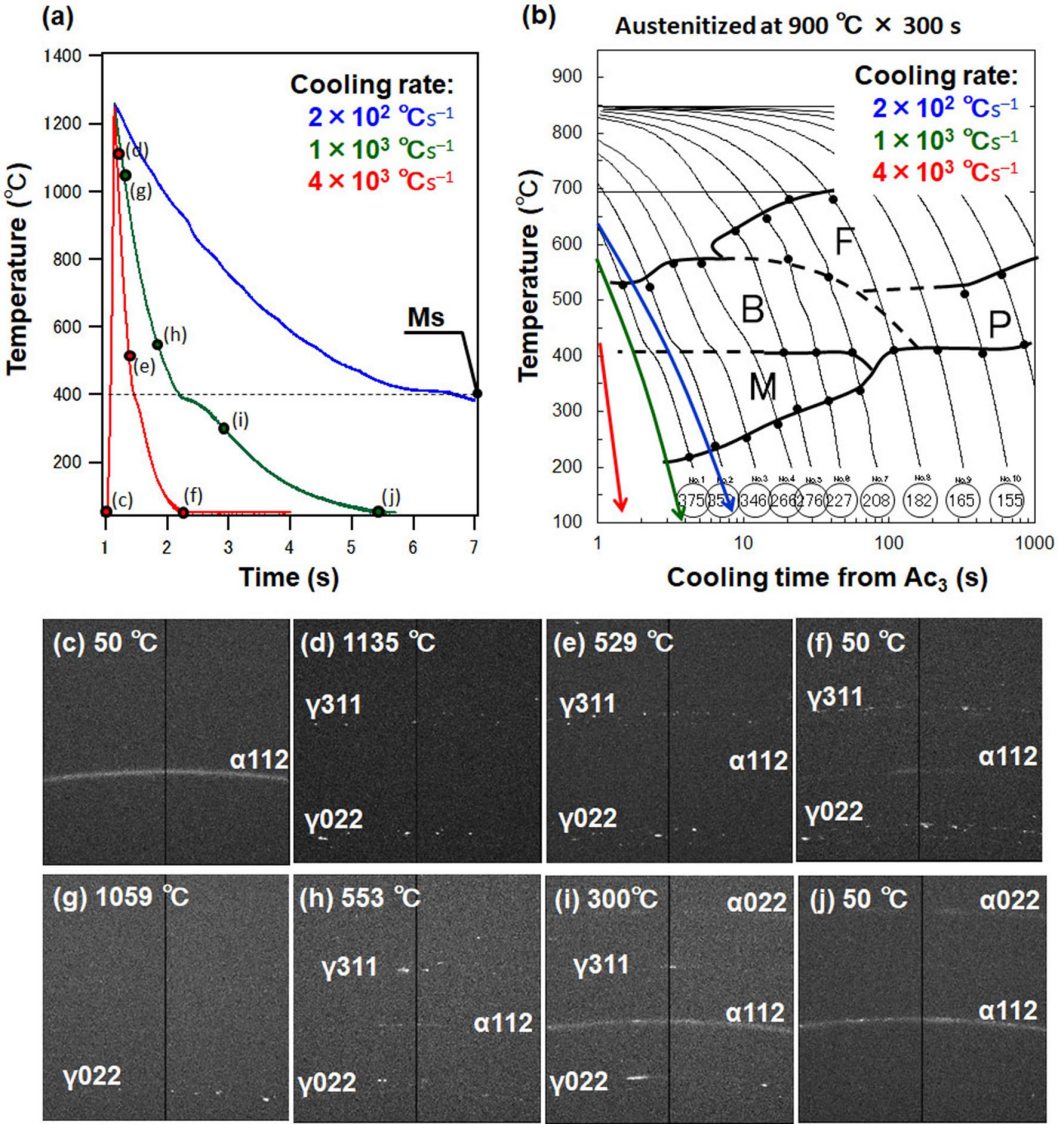
Cooling curve for a heating rate of 1.0 × 10^4^ °C s^–1^. (**a**) Cooling curve measured using a monochromatic pyrometer and (**b**) continuous cooling transformation diagram. M, B, F and P represent martensite, bainite, ferrite, and perlite, respectively. The circled numbers above the x-axis in (**b**) indicate the room temperature hardness. (**c**–**j**) examples of the two-dimensional diffraction pattern at temperatures of (**a**)**-**(**j**) in (**a**).

Figure 2a shows the temperature profile and the pre-prepared continuous cooling transformation (CCT) diagram of 0.1 mass% C–2 mass% Mn steel. High cooling rates of 1.0 × 10^3^ and 4.0 × 10^3^ °C s^−1^ were obtained by spraying a pressurized gas of 0.8 MPa immediately after the ultrafast heating to approximately 1200 °C at 1.2 × 10^4^ °C s^–1^ and controlling the distance between the gas nozzle and sample. According to the CCT diagram shown in Fig. 2b, at a lower cooling rate of 2.0 × 10^2^ °C s^−1^, a bainite-transformation starting temperature (Bs) of approximately 550 °C and a martensitic-transformation starting temperature (Ms) of approximately 400 °C were observed. At lower cooling rates, a Ms of approximately 400 °C was observed.

Some diffraction patterns under cooling are shown in Figs. 2c–j. The diffraction pattern of the γ phase looks spotty with crystal preferred orientation under cooling. Furthermore, the diffraction of the oxide was observed only slightly at 50 °C after cooling. At the cooling rate of 4.0 × 10^3^ °C s^−1^, the diffraction spots, γ022 and γ311, of the γ phase were observed at 50 °C after cooling. In contrast, at the cooling rate of 1.0 × 10^3^ °C s^−1^, no diffraction spots of the γ phase were observed at all.

Herein, at a heating rate of 1.2 × 10^4^ °C s^−1^, no surface oxide layer was formed as revealed by the results of previous research on ultrafast heating^16^. When cooling at 1.0 × 10^3^ and 4.0 × 10^3^ °C s^−1^, the surface oxide layer was subsequently suppressed by spraying helium gas. A slight diffraction peak was observed at a low angle, but the peak could be separated. On the other hand, it was difficult to analyze the results of the slow cooling process owing to the effects of the surface oxides at the lower cooling rate of 2.0 × 10^2^ °C s^−1^ without helium gas spraying. Therefore, we discuss the microstructure formation based on dislocation-density changes observed at the high cooling rates of 1.0 × 10^3^ and 4.0 × 10^3^ °C s^−1^.

**Figure 3:**
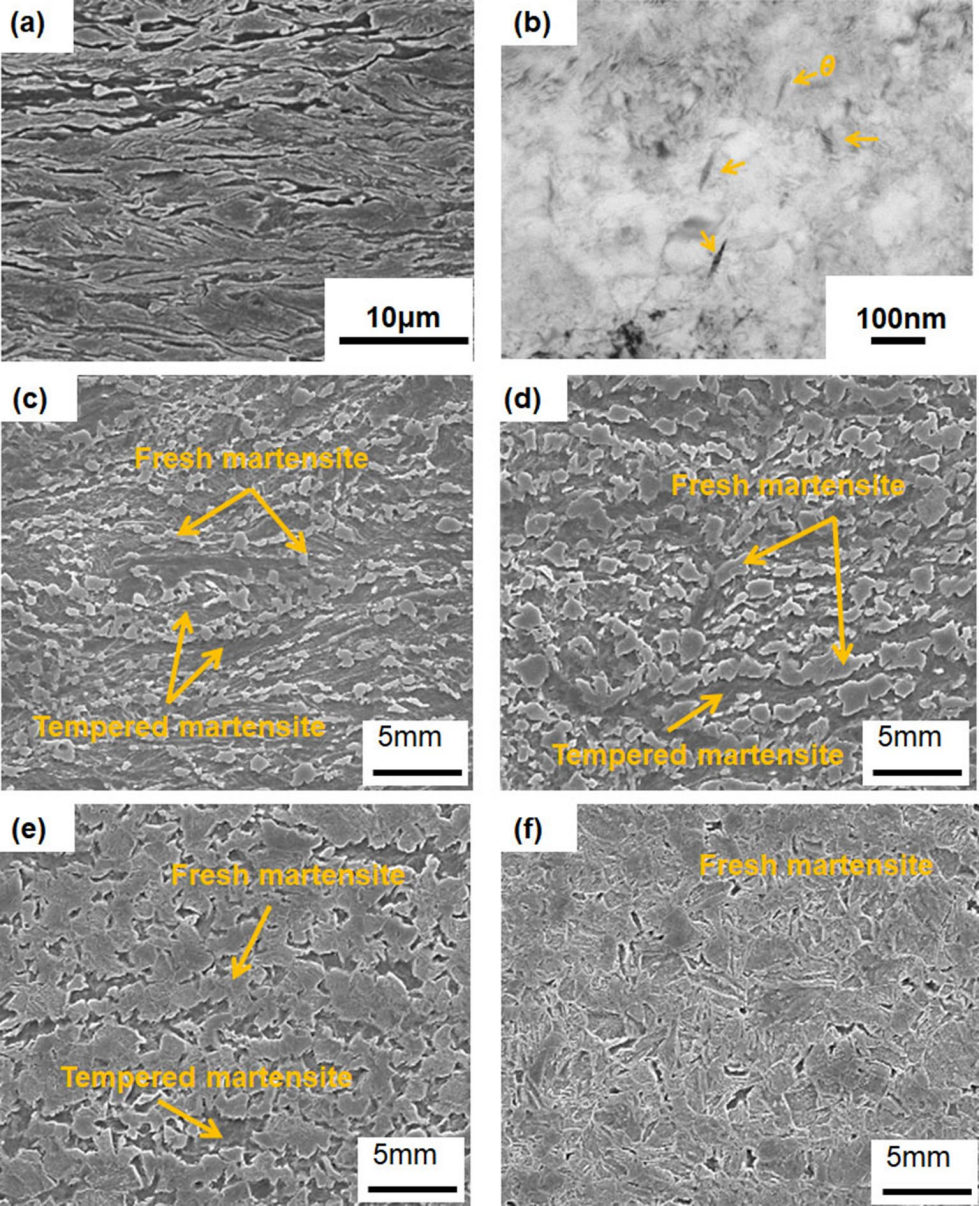
Quenched microstructure during ultrafast heating. (**a**) Initial microstructure and (**b**) θ-Fe_3_C in the initial microstructure. (**c**–**f**) show the microstructural change in the two phase zone at 760, 800, 845, and 876 °C.

As shown in Fig. 3a, the initial microstructure is tempered martensite, in which a small amount of fine θ-Fe_3_C was observed in, as shown in Fig. 3b. In the phase transformation process shown in Figs. 3c–f, the γ phase (fresh martensite) transforms uniformly from the martensitic phase (tempered martensite), at 876 °C, it is presumed to be full austenite. In addition, no Fe_3_C was observed even at 4.0 × 10^3^ °C s^−1^, as discussed below. This means that even with ultrafast heating of 1.2 × 10^4^ °C s^−1^, a small amount of Fe_3_C is dissolved when the temperature attains 1200 °C, and the starting conditions before cooling are the same.

### Phase transformation and dislocation density during ultrafast cooling

**Figure 4:**
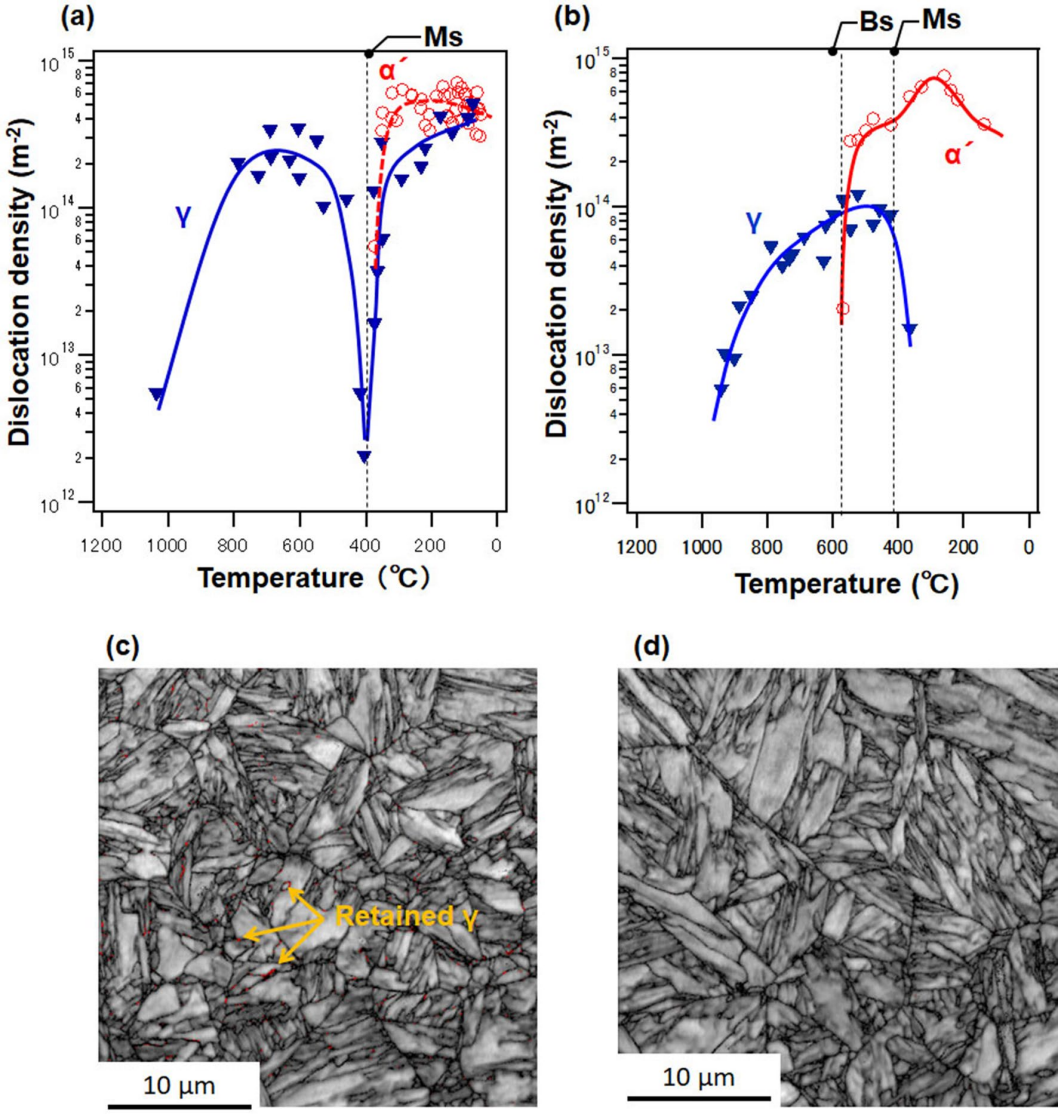
Dislocation density as a function of temperature at cooling rates and the image quality map of the EBSD after cooling off (**a**) and (**c**) 4.0 × 10^3^ and (**b**) and (**d**) 1.0 × 10^3^ °C s^–1^. The red area indicated by the arrow in (**c**) is the finely distributed γ-phase of less than several hundred nm.

Figure 4 shows the results of the dislocation-density analysis at cooling rates of (a) 4.0 × 10^3^ and (b) 1.0 × 10^3^ °C s^–1^. The diffraction signal of the α phase was observed at approximately 400 °C at a cooling rate of 4.0 × 10^3^ °C s^–1^. The transformation from the γ phase to the α phase at approximately 400 °C shows a martensitic (α′) transformation, as inferred from the CCT diagram in Fig. 2b. In other words, at the cooling rate of 4.0 × 10^3^ °C s^−1^, a martensitic transformation occurred without passing through the CCT nose of bainite.

Furthermore, the retained γ-phase was observed even at room temperature. The finely distributed γ-phase of less than several hundred nm at the grain boundary was also observed only after 4.0 × 10^3^ °C s^–1^ cooling test as shown in Fig. 4 (c) 4.0 × 10^3^ and (d) 1.0 × 10^3^ °C s^–1^ in the electron backscattering diffraction (EBSD) corresponding to the X-ray dislocation density analysis shown in Fig. 4a, b. The confidence index in the red region indicated by the arrow was greater than 0.14, which was reliable enough for γ-phase^37^.

This is because a high cooling rate results in a decrease in the Ms. The factors that lower the Ms point are an increase in the amount of carbon in the γ phase^38^, an increase in the dislocation density in the γ phase^39^, and the refinement of the γ grains^40–43^. Our previous report has shown that rapid heating increased the dislocation density in the high-temperature γ phase and formed the finer γ grains^16^. Since this dislocation density decreases after the formation of the γ phase, the shorter the holding time in the high-temperature region, the more the dislocation density is maintained. The γ-phase remains large and the Ms point is lowered because the cooling rate of 4.0 × 10^3^ °C s^–1^ has a shorter time than that of 1.0 × 10^3^ °C s^–1^ in the high-temperature region. Furthermore, since γ grains coarsen due to grain growth, the shorter the holding time in the high-temperature region, the finer the grains are maintained. Therefore, the Ms point is lowered at 4.0 × 10^3^ °C s^–1^ for relatively finer grains. It is well-known that when the γ grain size becomes smaller than 1 μm, the Ms point decreases^40–43^, and the γ grains produced by rapid heating and cooling are at a level of refinement.

The amount of the retained γ phase before and after the test was 0% and 0.4% at the cooling rate of 4.0 × 10^3^ °C s^−1^ by laboratories’ X-ray diffraction, respectively. Since the intensity of the XFEL differs with each pulse, the diffraction images at 35°, 55°, and 77° have different intensities. Therefore, it was difficult to analyze the phase fraction during the cooling process.

With the growing α′ phase, the dislocation density of the α′ and γ phases increased again. Based on in-situ neutron diffraction experiments, Christien et al.^44^ reported an increase in the dislocation density of α′ and γ phases after the onset of the transformation. Meanwhile, based on in-situ high-energy X-ray diffraction experiments, Macchi et al. ^45^ reported an increase in the dislocations due to the stresses on the surrounding α′ and γ phases caused by the growth of the α′ phase. Finally, near the room temperature, the decrease in the fraction of the screw dislocations could occur as a result of the rearrangement of the variants, the rearrangement of the dislocations due to the compression field caused by transformation stresses, and the stabilization of the dislocation substructure owing to the approximately halved fraction of the screw-to-edge dislocations, that, the dislocation loop approached a perfect circle. At approximately 50 °C, the dislocation densities of the retained γ and α′ phases were similar. In contrast, Fig. 3b shows the results of the dislocation density analysis at a cooling rate of 1.0 × 10^3^ °C s^−1^. The dislocation density of the γ phase increased during the cooling process from 1200 °C, and the diffraction signal of the α phase was observed at approximately 600 °C. The transformation from the γ phase to the α phase at approximately 600 °C indicated a bainitic transformation, as inferred from the CCT diagram in Fig. 2b. Upon further cooling, the dislocation density of the α phase further increased at approximately 400 °C, where the starting point of a martensitic transformation was considered. The dislocation density of the γ phase decreased with increase in the α′ phase from approximately 400 °C. At a cooling rate of 1.0 × 10^3^ °C s^−1^, the γ phase was not observed after the martensitic transformation.

The dislocation density of the γ phase increased during the cooling process at cooling rates of 4.0 × 10^3^ and 1.0 × 10^3^ °C s^−1^. Several factors might have contributed to the increase in the dislocation density of the γ phase up to the martensitic transformation during the cooling process^46^. In general, due to supersaturation or supercooling, atoms do not have sufficient time to align themselves in their normal positions and are, therefore, fixed in the wrong position, resulting in dislocations. In slow cooling, that is, slow crystal growth, crystals with a diameter of 1–100 μm often do not contain dislocations^47^. In addition, when local impurities bend the crystal lattice during crystal growth, dislocations are formed to reduce the strain energy. Furthermore, dislocation loops are formed when the vacancies clustered in the plate form collapse owing to decrease in the surface energy. Dislocations are formed at high temperatures, and thermal equilibrium vacancies increase exponentially with temperature, where supersaturated vacancies held in non-equilibrium via rapid cooling might be present. Researchers have reported that the interaction between screw dislocation components with point defects resulted in the helicalization of the dislocations, followed by the growth of the dislocation loops and the formation of vacancy loops in a short time^48–51^. Furthermore, Munday et al.^52^ reported the formation mechanism of prismatic and helical dislocation loops from defects. A recent in situ transmission electron microscope (TEM) nanoindentation analysis shows that the cross slip of the shear loop is favored, resulting in a transition from the prismatic dislocation loops and then to open half loops to helical dislocations as the indentation size increases^53^.

Subsequently, as shown in Eq. (5), it has been reported that the dislocation density *ρ* increases with increasing cooling rate *R*^54^.

5 $$ \rho = \rho_{0} \cdot exp\left( { - k/R} \right), $$

where *k* is a constant. Therefore, the increase in the dislocation density of the γ phase is dependent on the cooling rate.

### Momentary change in dislocation density

Immediately before the martensitic transformation, the dislocation density of the γ phase decreased once, as shown in Fig. 4. Although the considerable change in the dislocation density of the γ phase in this duration seems incomprehensible, the decrease in the dislocation density indicates the softening of the material. Equation (1), which is used to calculate the dislocation density, is a function of the dislocation density *ρ* and the contrast factor *C* (which in turn is a function of the elastic stiffness). In the dislocation density analysis, the elastic stiffness was constant at each temperature. However, the elastic compliance may be altered by factors other than temperature. Lattice softening^55,56^ is a precursor of thermoelastic martensitic transformation, typically known as phonon softening^57^. In thermoelastic martensite, where the transformation temperature is approximately room temperature, phonon softening is often observed as a transformation precursor^58–60^. Neuhaus et al.^61^ reported the occurrence of lattice softening in the α → γ inverse transformation of pure iron during heating. In addition, Fujita et al.^62^ suggested that the intermediate phases observed during the FCC → BCC transformation in Fe–Mn–C steel are likely to undergo lattice softening along [11-2]/(111). Meanwhile, phonons were measured directly via inelastic neutron scattering in the γ phase; however, no significant anomalies were observed because the measurement temperature differed from the transformation temperature^63^. No phonon softening was observed in the cooling process of steel owing to the high transformation temperature. However, the lattice softening observed in the low-carbon steels used in this study could change the elastic constants and an apparent decrease in the dislocation density. If the dislocation density *ρ* in Eq. (1) is maintained, then the contrast factor *C* decreases—this signifies the softening of the phonon. This result suggests that carbon steels that transform at high temperatures can exhibit the phenomena observed in thermoelastic martensite.

The phonon softening of the FCC-γ matrix may decrease the stacking fault energy of the BCC-α (or intermediate HCP) embryos. The stacking fault energy *γ* of an ellipsoidal embryo can be estimated using an expression by Olson^63,64^, as shown in Eq. (6):

6 $$\gamma =n{\rho }_{A}\left(\Delta {G}^{chem}+{E}^{str}\right)+2\sigma \left(n\right),$$

where $n$ is the atomic plane thickness; ${\rho }_{A}$ is the density of the atoms in a closed packed plane; $\Delta {G}^{chem}$ and ${E}^{str}$ are the chemical-free energy difference and coherency strain energy, respectively, and $\sigma \left(n\right)$ is the embryo/matrix interfacial energy. The value of $\gamma$ decreases with the increase in the embryo size. This implies that the embryo grows spontaneously with a critical thickness (critical size) ${n}^{*}$, where $\gamma \le 0$. Olson estimated the value of a Fe–Ni alloy as ${n}^{*}=13.5$. If phonon softening occurs in the FCC-γ matrix, then ${E}^{str}$ decreases, and ${n}^{*}$ decreases to approximately 8.1^64,65^. This renders the thermally activated embryo stable and increases the nucleation rate at or below the Ms, thereby resulting in a significant number of nuclei in the matrix. Some alloys (Fe_3_Pt^66^, Fe–Pd^67^, and Ti–44 at.% Ni–6 at.% Fe^68^) exhibit phonon softening because nesting vectors in the electronic structure exhibit tweed structures (pre-martensite) measuring several tens of nanometers at temperatures slightly above the Ms. In particular, Ti–44 at.% Ni–6 at.% Fe assumes this nanometer-sized tweed structure below the incommensurate-commensurate phase-transformation (stain-glass) temperature^69^. Fe–C martensitic steels exhibit a fine microstructure only if the coarsening of the BCC-α (or intermediate HCP) nuclei is inhibited during cooling at room temperature. An increase in the dislocation density of the high-temperature γ phase due to ultrafast heating and cooling might have facilitated softening in the steel.

### Momentary change in dislocation characteristics

**Figure 5:**
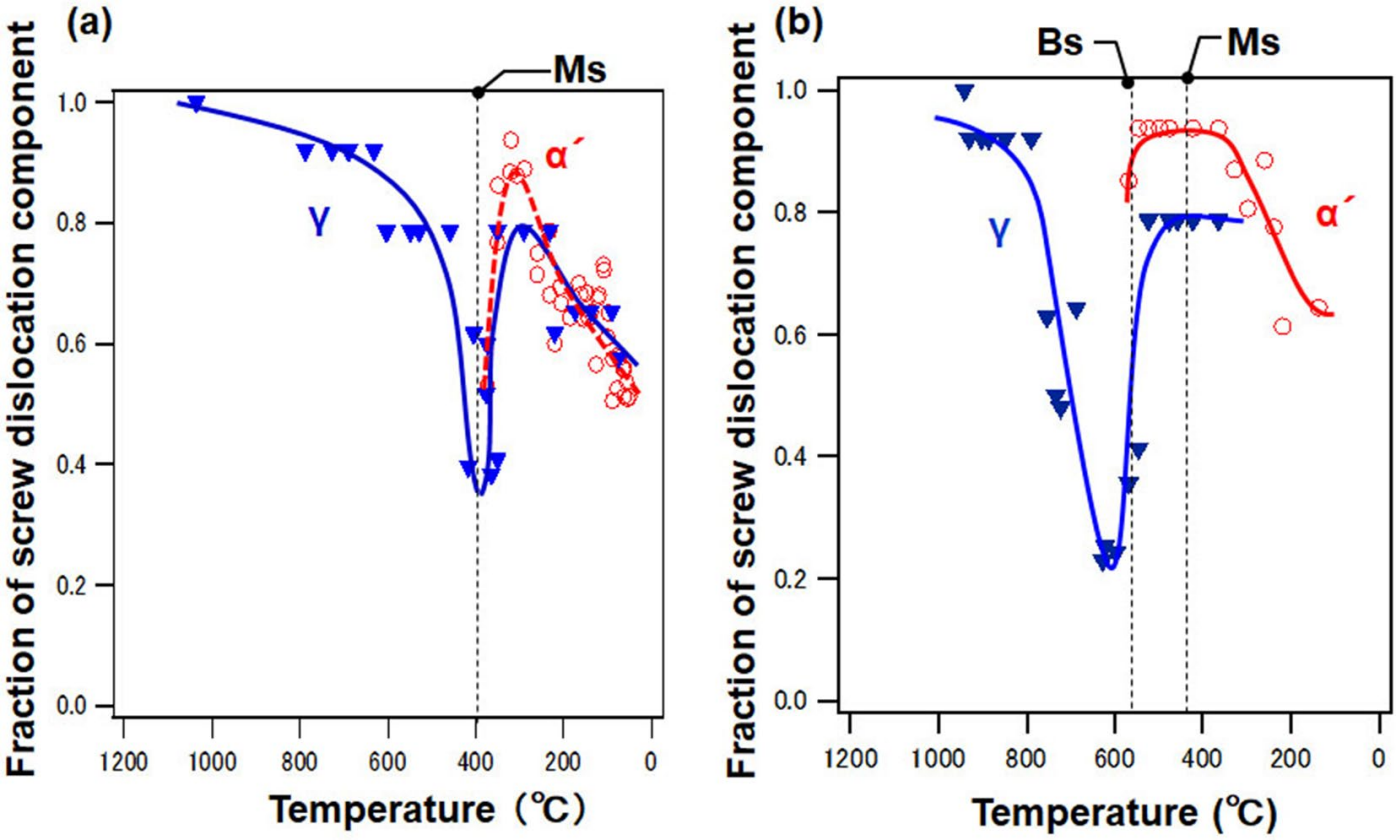
Dislocation characteristics as a function of temperature at cooling rates of (**a**) 4.0 × 10^3^ and (**b**) 1.0 × 10^3^ °C s^–1^.

Figure 5 shows the change in the dislocation characteristics at cooling rates of 4.0 × 10^3^ and 1.0 × 10^3^ °C s^−1^. A considerable change in the dislocation characteristics in the γ phase appeared in a short duration (approximately 0.15 s) immediately before the FCC-γ to BCC-α′ phase transformation. This phenomenon was a momentary change in the dislocation substructure captured via femtosecond observations. This phenomenon suggests that the phase transformation of low-carbon steel occurred under ultrafast cooling, as proposed by Sherrby et al.^70^ and numerous studies based on the Engel–Brewer electron theory^71^. HCP formation requires a partial Shockley dislocation to extend the dislocation.

**Figure 6:**
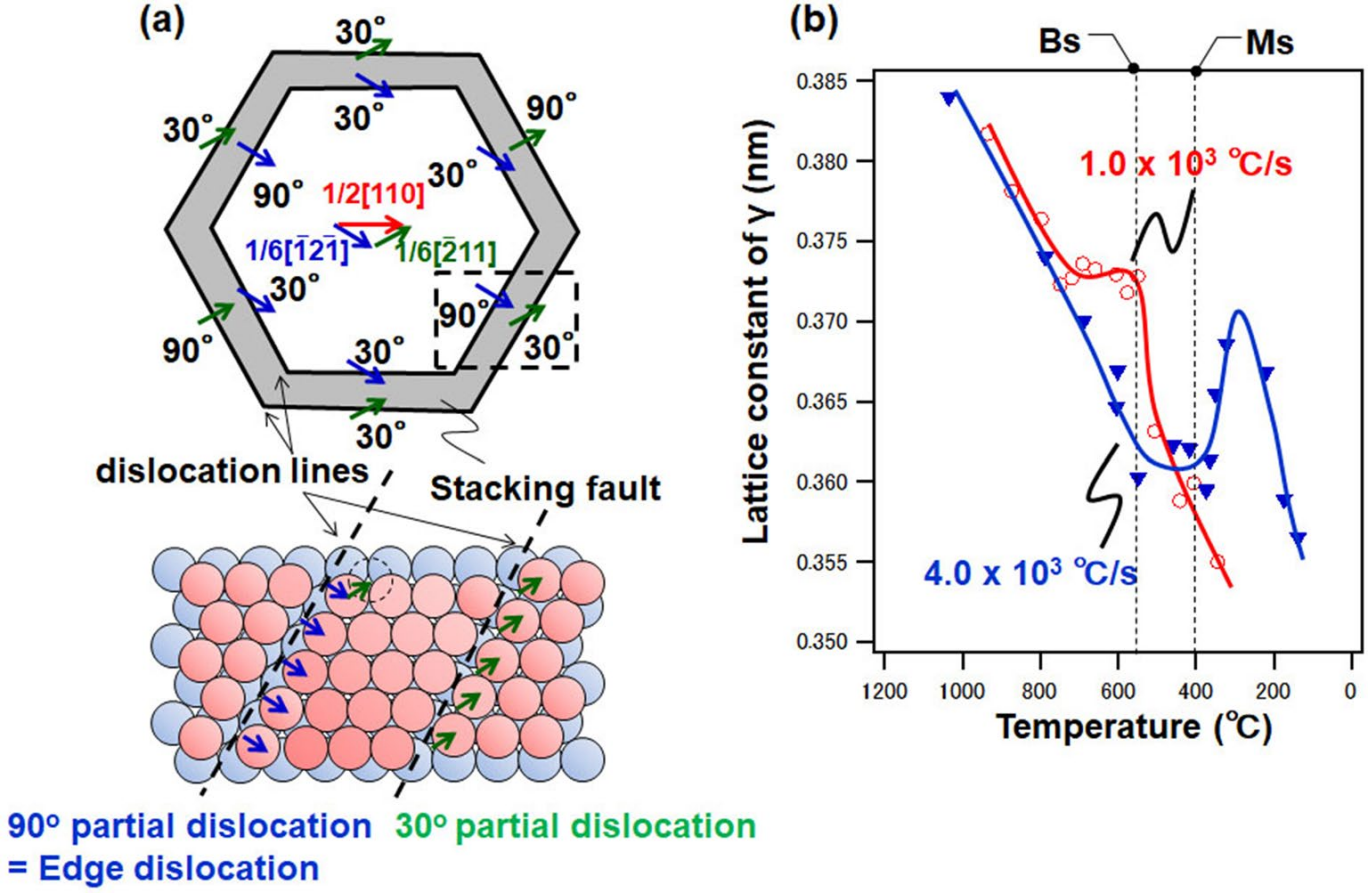
Schematic illustration of the dislocation loop. (**a**) Dislocation loop model of {111} <110>. (**b**) Temperature dependence of γ phase lattice constant at a cooling rate of 1.0 × 10^3^ °C s^–1^ (red line) and 4.0 × 10^3^ °C s^–1^ (blue line).

Figure 6a shows a model of the {110} linear dislocations of the FCC with a simplified dislocation loop. The dislocation loop comprised a screw dislocation parallel to the Burgers vector and a 60° dislocation. As the dislocation line expanded, the helical dislocation component decomposed into two 30° partial dislocations, and the 60° dislocation decomposed into 30° and 90° partial dislocations. Since the 90° partial dislocation was perpendicular to the dislocation line, it was, therefore, considered an edge dislocation. It can be assumed that this dislocation expansion resulted in an increase in the edge dislocation component and a decrease in the screw dislocation component^72^. In addition, it was inferred that many dislocation extensions or stacking faults were formed before the γ → α phase transformation. Bogers and Burgers^73^ speculated that the shear required for the phase transformation is a Shockley partial dislocation, and that the transformation can proceed by the migration of a screw dislocation. Meanwhile, Lagneborg^74^ reported that the martensite-like region near the center of the Shockley partial dislocation is a preferential nucleation site. Furthermore, Venables^75^ reported that the α′ phase is nucleated from the HCP-ε phase and grows via the migration of screw dislocations.

As shown in Eq. (7), an increase in the dislocation density is associated with an increase in the stacking fault probability *f*^76^.

7 $$\rho =3.27\times {10}^{14}\cdot f+8.48\times {10}^{12}$$

Furthermore, as shown in Fig. 4b, the lattice parameter of the γ phase may have expanded immediately before the bainitic transformation, resulting in the formation of the HCP phase. In 18 mass % Cr–8 mass % Ni stainless steel, the BCC-α′ and HCP-ε phases were formed during the transformation. The ε phase was formed by the propagation of Shockley’s partial dislocation, which introduced stacking faults^77^. Kato et al.^78^ reported the motion of the <111> {112} shear system in the early stages of the martensitic transformation, and that stacking faults and the intermediate phase formation of HCP structures were crucial for facilitating the γ → α phase transformation. Based on the TEM analysis, it was observed that the HCP phase occurred in Fe–Si–C steel, and that stacking faults were introduced immediately before the bainitic transformation^79^. An FCC to BCC transformation model via an HCP structure has been proposed for martensitic transformation in low-carbon steels^80^. In other words, the transformation from the γ phase to α′ phase is (i) either an ε phase generation or a defective α′ phase generation with the defective ε phase as an intermediate phase, (ii) a direct transformation, (iii) or a transformation to the α′ phase through a defective γ phase. The same behavior may occur in the bainite transformation, which is inferred to be a transient phenomenon that occurs in the transformation from the γ phase to the α phase.

As shown in Fig. 6b, the lattice-parameter change of the γ phase at a cooling rate of 1.0 × 10^3^ °C s^–1^ was delayed without thermal shrinkage during cooling, that is, lattice expansion occurred immediately before the BCC-α transformation. Although it is still to be clarified at 4.0 × 10^3^ °C s^–1^, it might constitute a delay in the lattice parameter before the martensitic transformation. That is, it is suggested that the lattice expansion occurs just before the first FCC (γ) → BCC (α or α′) transformation during cooling. However, the lattice constant of the fine γ phase also gradually expands due to the tensile stress caused following the martensitic transformation. The subsequent decrease in temperature causes the strain to relax along with the rearrangement of the dislocations in the α′ phase, and then the lattice parameter of the γ phase also decreases as well. In a detailed analysis of austenitic steels, Sahu et al.^81^ reported that the transformation of FCC-γ to HCP-ε during cooling results in a decrease in lattice volume via an HCP structure, resulting in the lattice expansion of the FCC structure. As described above, the dislocation density in the γ phase increased from 10^12^ to 10^14^ m^-2^, and the lattice constant decreased monotonically, although lattice expansion occurred immediately before the Ms, which was essentially an elastic shrinkage. In other words, the increase in the dislocations of the γ phase was not a result of plastic deformation under thermal stress. Because thermal stress is a function of temperature, the amount of thermal expansion and shrinkage is the same for different cooling rates. In contrast, as the martensitic transformation progresses, self-relaxation causes the slip to spread. The dislocations of the α′ phase are introduced by this slip deformation and increase due to the martensitic transformation.

The decrease in dislocations below 300 °C in Fig. 4b is caused by the decrease, which means coalescence and annihilation, of screw dislocations as shown in Fig. 5b. The decrease in the number of screw dislocations results from the cross slip occurrence even at relatively low temperatures below 400 °C, and the dislocations in the different slip planes can coalesce and annihilate relatively easily by cross slip. In contrast, edge dislocations require atomic diffusion, so that the dislocations can coalesce and annihilate each other due to climb motion only at high temperatures.

Finally, at the cooling rate of 1.0 × 10^3^ °C s^-1^, the fraction of the screw dislocation component decreases at approximately 600 °C as shown in Fig. 5b, while the dislocation density increases as shown in Fig. 4b. We believe that the change in the total dislocation density could be separated from the change in the fraction of the dislocation character. The change in the total dislocation density occurs as a precursor to the martensitic transformation as mentioned above. In contrast, the change in the dislocation character occurs in the first FCC (γ) → BCC (α or α′) phase transformation during the cooling process. Considering the dislocation loop model shown in Fig. 6a, it can be assumed that the change in the dislocation character is related to the formation of HCP, that is, the introduction of the stacking defects. In other words, the increase in the number of edge dislocations means the decrease in the number of screw dislocations, and ideally, the total dislocation density is maintained even if the fraction of the dislocation character changes subsequently. At 4.0 × 10^3^ °C s^−1^, the martensitic transformation is the first FCC (γ) → BCC phase transformation without a Bs. Therefore, the decrease in the dislocation density and fraction of the screw dislocation component occur simultaneously.

### Formation of fine microstructures

**Figure 7:**
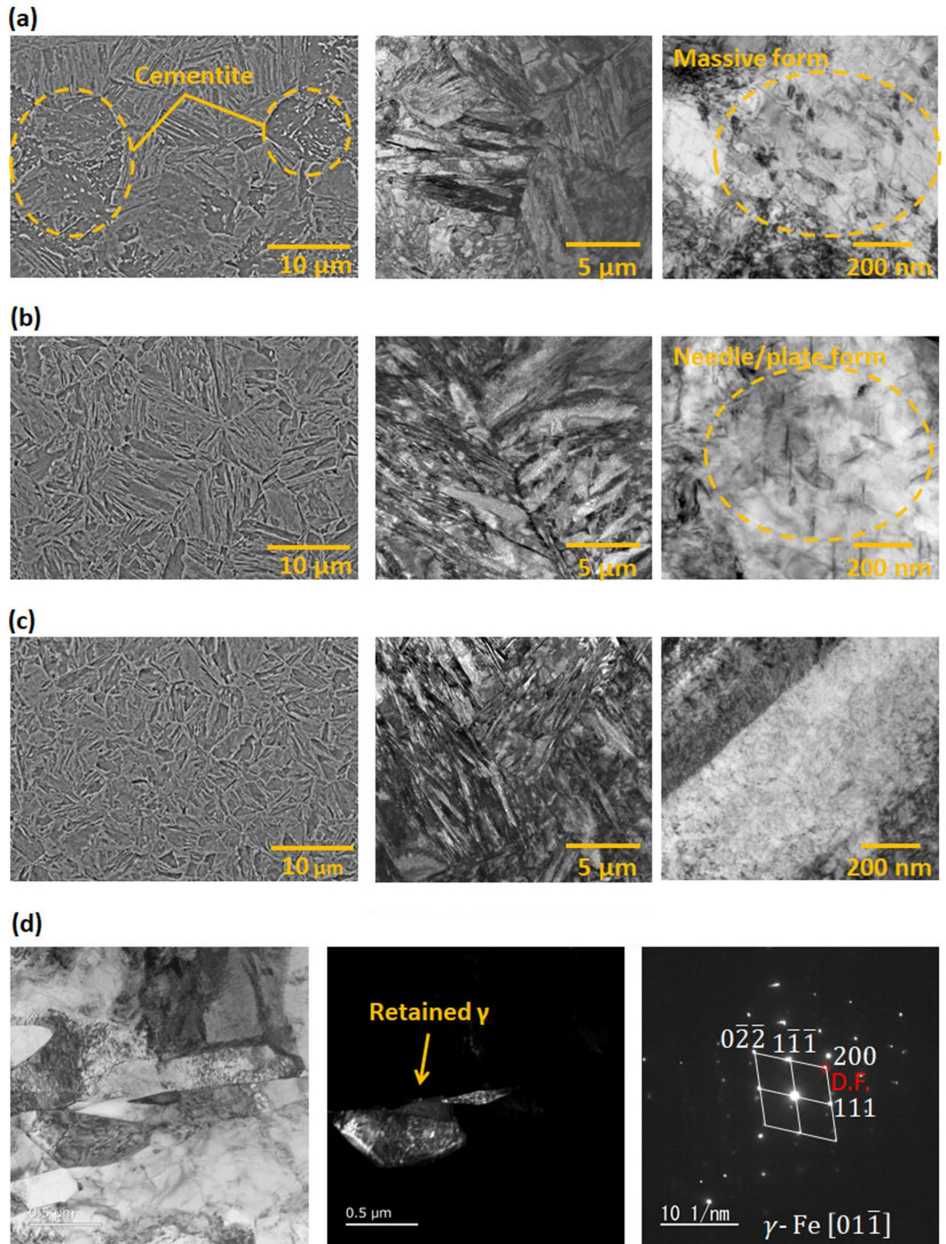
SEM images (left) and TEM images (center and right) of microstructures obtained by gas-spraying cooling at rates of (**a**) 2.0 × 10^2^, (**b**) 1.0 × 10^3^, and (**c**) 4.0 × 10^3^ °C s^–1^ after heating up to approximately 1200 °C at a rate of 1.0 × 10^4^ °C s^–1^. (**d**) TEM images of the residual γ-phase with a cooling rate of 4.0 × 10^3^ °C s^–1^, from left to right: bright-field image, (200) dark field image, and diffraction pattern.

The short-range diffusion transformation at a high cooling rate resulted in the formation of a fine microstructure without cementite (Fe_3_C), as shown in Fig. 7. At 2.0 × 10^2^ °C s^−1^, a coarsened microstructure exhibiting massive cementite was formed. At 1.0 × 10^3^ °C s^−1^, a fine microstructure exhibiting needle-shaped cementite was formed^82^. The microstructure can be categorized as auto-tempered martensite^83^. At 4.0 × 10^3^ °C s^−1^, a relatively finer microstructure, which indicates a full martensite, was formed without Fe_3_C, that is, a solid carbon solution. Therefore, Fe_3_C was observed, which is precipitated during cooling, at the cooling rate of 2.0 × 10^2^ °C s^-1^ and 1.0 × 10^3^ °C s^-1^. As shown by the TEM images in Fig. 7 (d), the retained γ phase, which exists at several hundred nm, appears to have a high density of dislocations like a micro band in austenite^84^. This result also corresponds to the EBSD result shown in Fig. 4c. This proved and supported our claims.

We successfully captured the γ → α phase transformation and its transformation kinetics during the ultrafast cooling. The fine γ grains, which were formed via ultrafast heating, did not undergo Ostwald growth and precipitation owing to the insufficient time due to the ultrafast cooling. In contrast, the dislocation density increased significantly in the γ grain. The fine microstructure of the full martensite resulted in fine γ grains and a high dislocation density in the γ grain. Hence, it was inferred that the fine γ phase formed by ultrafast heating increased the dislocation density (elastic strain energy) during ultrafast cooling, resulting in microstructural refinement.

## Conclusions

In this study, a new in situ XFEL measurement technique integrated with an XLPA was developed and applied in the field of steel science for the first time. We performed operand XRD measurements of Fe–0.1 mass% C–2 mass% Mn transforming from γ to α′ during ultrafast cooling at rates of 1.0 × 10^3^ and 4.0 × 10^3^ °C s^–1^ after ultrafast heating at rates exceeding 10^4^ °C s^–1^. The dynamic changes in the dislocation densities were successfully evaluated on a timescale shorter than 1 s during the ultrafast cooling.

The dislocation density is a key parameter in the production of high-strength steels. High dislocation densities occurred at high heating rates to produce fine-grained crystals. The dislocation density was further multiplied by the martensitic transformations with the ultrafast cooling. This study demonstrated that by directly observing the dynamic changes in the dislocation density, the kinetics of the microstructural changes occurring under steep thermal gradients could be comprehended more effectively. An ultrafast cooling process following ultrafast heating was observed for the first time in this study. Such observations could facilitate the further development of functional steels and new manufacturing processes for fine microstructure formation in low-carbon steels from the perspective of dislocation density. Since 1970, when researchers began studying thermomechanical control processes, ultrafast XRD with XFELs have resulted in significant breakthroughs in advanced metallurgy, including the novel research method reported herein, which can be applied to various alloy systems that exhibit electrical conduction other than Fe–C steel.
